# Dataset of microscopic images and infrared spectra of beach sediment samples from Juara, Salang, and Tulai in Tioman Island, Malaysia

**DOI:** 10.1016/j.dib.2025.111907

**Published:** 2025-07-20

**Authors:** Mohd Rashidi Abdull Manap, Muhammad Ibadurrahman bin Imizan, Jannik Werner Dams, Norizah Abdul Rahman, Fathin Ayunni Zaiffira Waini, Nur Hayatna Mukhni, Wan Lutfi Wan Johari

**Affiliations:** a Department of Chemistry, Faculty of Science, Universiti Putra Malaysia, 43400 Serdang, Selangor, Malaysia; b Medical Microspectroscopy Research Group, Department of Experimental Medical Science, Lund University, 22180 Lund, Sweden; c School of Biological Sciences, University of Manchester, Oxford Rd, Manchester M13 9PL, United Kingdom; d Department of Geography, Rheinische Friedrich-Wilhelms-Universität Bonn, Regina-Pacis-Weg 3, 53113 Bonn, Germany; e Department of Environmental Science and Technology, Faculty of Forestry and Environment, Universiti Putra Malaysia, 43400 Serdang, Selangor, Malaysia

**Keywords:** ATR-FTIR, Malaysia, Microplastic, Microscopic images, Spectra

## Abstract

This dataset presents a comprehensive collection of microscopic images, infrared (IR) spectra of particles obtained from beach sediment in Tioman Island, Malaysia. Sediment samples were collected from multiple sites across three selected beaches to identify material composition and assess the concentration of plastic particles, including both microplastics (<5 mm) and macroplastics (>5 mm), in the region. The IR spectroscopic analysis was carried out in the mid-infrared range (4000–370 cm⁻¹), enabling detailed vibrational characterization of compounds present in the samples. By integrating multiple spectroscopic techniques and vibrational calculations, this dataset provides a valuable resource for environmental monitoring, microplastic pollution studies, and material identification in marine ecosystems.

Specifications Table

**Table:** 

Subject	Analytical Chemistry, Spectroscopy
Specific subject area	Chemical characterisation of marine sediment particles
Type of data	Raw and analyzed data includes table, ATR-FTIR spectra, microscopic images, map images, and sampling images.
Data collection	Micro- and macroparticles were extracted from beach sediment samples through sieving, density separation, and subsequent microscopic and spectroscopic analysis. Sediments were sieved using stainless-steel mesh sieves (5.60 mm, 710 µm, 212 µm) to isolate particles. Separated fractions underwent density separation using clean seawater. Recovered particles were filtered and visually categorized by type, color, and size. Microscopic images were captured with an Olympus CX33 transmitted light microscope (4 × objective, camera-connected). Polymer identification was performed using a Shimadzu IRAffinity-1S FTIR spectrophotometer with ATR accessory, scanning from 4000 to 370 cm⁻¹ at 4 cm⁻¹ resolution, averaged over 32 scans in transmittance mode.
Data source location	Latitude and longitude and GPS coordinates, for collected samples/data: Juara Beach S01- 2°47′54.1″N 104°12′24.5″E S02- 2°47′44.9″N 104°12′15.0″E S03- 2°47′28.9″N 104°12′10.4″E S04- 2°47′15.5″N 104°12′13.0″E S05- 2°47′02.8″N 104°12′15.5″E S06- 2°46′49.1″N 104°12′22.8″E Tulai Beach S01- 2°54′22.7″N 104°06′02.8″E S02- 2°54′22.8″N 104°06′00.9″E S03- 2°54′24.6″N 104°05′59.6″E S04- 2°54′26.6″N 104°05′58.2″E S05- 2°54′28.0″N 104°05′56.5″E Salang Beach S01- 2°52′37.7″N 104°09′17.3″E S02- 2°52′34.9″N 104°09′15.9″E S03- 2°52′32.2″N 104°09′13.9″E S04- 2°52′29.2″N 104°09′11.6″E S05- 2°52′27.1″N 104°09′08.7″E Institution: Department of Chemistry, Faculty of Science, Universiti Putra Malaysia, Malaysia. Country: Malaysia Institution: Medical Microspectroscopy Research Group, Department of Experimental Medical Science, Lund University, 22,180 Lund, Sweden. Country: Sweden Institution: Research Institute for Applied Mechanics, Kyushu University, Kasuga, Japan Country: Japan
Data accessibility	Repository name: Manap, Mohd Rashidi Abdull (2025), “Supporting Dataset of FTIR, Microscopic Images, Sample Descriptions, and Study Map for Microplastic Analysis from Juara, Salang, and Tulai, Tioman Island, Malaysia (August 2024)”, Mendeley Data, V1 Data identification number: 10.17632/v4z92mndjf.1 Direct URL to data: https://data.mendeley.com/datasets/v4z92mndjf/1 Instructions for accessing these data: Click on the direct URL to obtain raw data.

## 1 Value of the Data

This dataset combines spectroscopic data with multivariate statistical analyses to support identification, classification, and quantification of microplastic particles smaller than 5 mm, with sizes ranging from 1.02 mm to 4.14 mm:

• **Microplastic concentration quantification**
  Enables the assessment of microplastics in beach sediments, providing insights into pollution levels.
• **Characterization of physical and synthetic attributes**
  Incudes size, color, shape, and polymer type variations supporting environmental forensics and oceanographers by linking these attributes (e.g., fiber vs. fragment) to potential sources and degradation pathways.
• **Color variation analysis**
  Facilitates source tracking and helps understand the effects of environmental weathering.
• **Integration with environmental variables (e.g., ocean currents)**
  When used alongside ocenographic data, this dataset helps explain spatial microplastic distribution patterns.
• **Synthetic polymer identification (e.g., polyethylene, polypropylene, polystyrene)**
  Provides chemical classification of particles, enhancing microplastic identification and monitoring.

## 2 Background

Microplastics are increasingly studied across a range of environmental samples including water, sediments, soil, air and biota for their distribution, characteristics, and ecological risks. In liquid state, both marine and freshwater samples are collected using methods like manta trawls and bulk water sampling, the latter being more sensitive to smaller particles [[1], [2], [3], [4], [5]]. Sediment samples from coasts and riverbanks require pretreatment steps such as digestion and density separation to isolate microplastics from organic debris [[6], [7], [8], [9], [10]]. Terrestrial assessments involve soil samples processed similarly, while airborne microplastics are captured on filters for spectroscopic identification [[10], [11], [12]]. Biotic monitoring reveals microplastic ingestion in aquatic organisms and land animals, contributing to our understanding of trophic transfer and biological accumulation [8,13]. Synthetic sources, such as microbeads in personal care products are also quantified for their environmental impact [[14], [15], [16]]. Analytical tools, including infrared and Raman spectroscopy, Py-GC–MS, and microscopy, are central to polymer identification and quantification [9,[17], [18], [19], [20], [21], [22], [23], [24], [25], [26]].

Characterizing microplastics in beach sediments is vital for understanding their ecological impact, tracing pollution sources, and guiding effective waste management. Such assessments help reveal how microplastics infiltrate marine ecosystems, harm aquatic organisms, and potentially threaten human health through food chain accumulation [[27], [28], [29], [30]]. Analyzing physical attributes and polymer types, such as polyethylene (PE), polypropylene (PP), polystyrene (PS), and polyester (PET), also aids in pinpointing pollution origins, whether from industrial discharge or consumer waste [[27], [28], [29], [30], [31]].

Microplastic datasets provide critical baselines for long-term environmental monitoring and evaluating the success of mitigation efforts [[30], [31], [32]]. In part of Southeast Asia, several regional studies of Malaysia from Miri to Terengganu have documented varying microplastic abundance linked to tourism, urbanization, and human activity [33]. For instance, higher concentrations were reported in tourist-heavy Kuala Langat [28], while turtle nesting beaches in Terengganu revealed concerning fiber presence, raising alarms about impacts on marine wildlife [34]. Collectively, these findings underscore the urgent need for continued monitoring of microplastics across coastal regions.

This study aims to analyze 35 plastic samples of various forms, colors, and sizes collected in August 2024 from 16 sites across three beaches in Tioman Island—Tulai Beach, Salang Beachand Juara Beach. The primary objective is to determine the concentration of microplastics at these sites, with a strong emphasis on material identification using infrared (IR) spectroscopy. Although the focus is on microplastics, particles exceeding 5 mm in size (macroplastics) were also recorded and included in the dataset to offer a more holistic view of plastic pollution in the area.

## 3 Data Description

This section presents the physical characterization of microplastic particles, including both raw and FTIR-analyzed results, along with microscopic images.

Table 1 provides detailed descriptions of the particles recovered from beach sediment samples, covering sample presence, separation method, physical characteristics (type, size, and color), and the geographical location of collection. Each sample was assigned a unique code following the format: BEACH_SITE_POINT_SEPARATION_DIGIT (e.g., JB_S01_B_F_1).

**Table 1:** Sample description of 35 particles analyzed in this study.

Beach	Site	Point	Separation	Type	Color	Sample code	Size	
							<5mm	>5mm
JB	S01	A	F			Absent		
			L			Absent		
			M			Absent		
			S			Absent		
		B	F	Fragment	White	JB_S01_B_F_1	3.23	
			L			Absent		
			M			Absent		
			S			Absent		
	S02	A	F			Absent		
			L			Absent		
			M	Fragment	Yellow	JB_S02_A_M_1	1.97	
			S			Absent		
		B	F			Absent		
			L			Absent		
			M			Absent		
			S			Absent		
	S03	A	F			Absent		
			L			Absent		
			M			Absent		
			S			Absent		
		B	F			Absent		
			L			Absent		
			M			Absent		
			S			Absent		
	S04	A	F			Absent		
			L			Absent		
			M			Absent		
			S			Absent		
		B	F			Absent		
			L			Absent		
			M			Absent		
			S			Absent		
	S05	A	F	Fragment	Yellow	JB_S05_A_F_1	2.82	
			L			Absent		
			M	Fragment	Green	JB_S05_A_M_1		6.00
			S			Absent		
		B	F			Absent		
			L			Absent		
			M	Fragment	Opaque	JB_S05_B_M_1		6.00
			S			Absent		
	S06	A	F			Absent		
			L			Absent		
			M			Absent		
			S			Absent		
		B	F			Absent		
			L			Absent		
			M	Fragment	Opaque	JB_S06_B_M_1	2.33	
			S			Absent		

TB	S01	A	F	Fragment	Yellow	TB_S01_A_F_1		5.50
				Fragment	Yellow	TB_S01_A_F_2		7.00
				Fragment	Yellow	TB_S01_A_F_3		7.50
				Fragment	White	TB_S01_A_F_4	3.80	
				Fragment	Green	TB_S01_A_F_5		32.00
			L			Absent		
			M			Absent		
			S			Absent		
		B	F	Fragment	White	TB_S01_B_F_1	2.60	
				Fragment	Yellow	TB_S01_B_F_2	2.64	
			L			Absent		
			M			Absent		
			S			Absent		
	S02	A	F			Absent		
			L			Absent		
			M	Fragment	Orange	TB_S02_A_M_1	1.30	
				Fragment	Purple	TB_S02_A_M_2	1.13	
			S			Absent		
		B	F			Absent		
			L			Absent		
			M	Fragment	White	TB_S02_B_M_1		5.10
			S			Absent		
	S03	A	F			Absent		
			L			Absent		
			M	Fragment	Orange	TB_S03_A_M_1	1.41	
			S			Absent		
		B	F			Absent		
			L			Absent		
			M			Absent		
			S			Absent		
	S04	A	F	Fragment	White	TB_S04_A_F_1	2.32	
				Fragment	Green	TB_S04_A_F_2	3.95	
				Fragment	White	TB_S04_A_F_3	1.54	
			L			Absent		
			M			Absent		
			S			Absent		
		B	F	Fragment	Red	TB_S04_B_F_1	1.46	
			L			Absent		
			M	Fragment	Orange	TB_S04_B_M_1	1.56	
			S			Absent		
	S05	A	F	Fragment	Purple	TB_S05_A_F_1		17.00
				Fragment	Red	TB_S05_A_F_2	3.35	
			L			Absent		
			M			Absent		
			S			Absent		
		B	F	Fragment	White	TB_S05_B_F_1	3.88	
				Fragment	Brown	TB_S05_B_F_2	4.00	
				Fragment	Yellow	TB_S05_B_F_3	3.95	
				Fragment	Yellow	TB_S05_B_F_4	1.75	
			L			Absent		
			M			Absent		
			S			Absent		

SLB	S01	A	F			Absent		
			L			Absent		
			M	Fragment	White	SLB_S01_A_M_1	4.00	
			S			Absent		
		B	F			Absent		
			L			Absent		
			M			Absent		
			S			Absent		
	S02	A	F	Fragment	White	SLB_S02_A_F_1		7.00
			L			Absent		
			M			Absent		
			S			Absent		
		B	F			Absent		
			L			Absent		
			M			Absent		
			S			Absent		
	S03	A	F			Absent		
			L	Fragment	Blue	SLB_S03_A_L_1		10.50
			M	Fragment	Red	SLB_S03_A_M_1	1.58	
			S	Fragment	Blue	SLB_S03_A_S_1	1.02	
		B	F			Absent		
			L			Absent		
			M			Absent		
			S			Absent		
	S04	A	F			Absent		
			L			Absent		
			M			Absent		
			S			Absent		
		B	F			Absent		
			L	Film	White	SLB_S04_B_L_1	4.14	
			M			Absent		
			S			Absent		
	S05	A	F			Absent		
			L			Absent		
			M			Absent		
			S			Absent		
		B	F			Absent		
			L			Absent		
			M			Absent		
			S	Fragment	Green	SLB_S05_B_S_1	1.77	

***Note:*** Each beach sediment particle is labeled according to its sampling site (e.g., JB_S01) and visual category. JB, TB, and SLB refer to Juara Beach, Tulai Beach, and Salang Beach, respectively. The letters F, L, M, and S indicate floating, large, medium, and small particles, respectively, which were separated and collected through density separation and sieving. The term “absent” in the sample code indicates that no microparticles were recovered from the corresponding beach sediment sample during these processes.

**Fig. 1:**
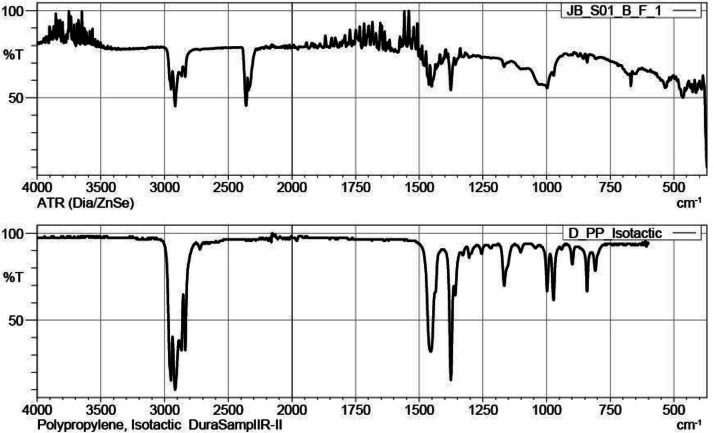
FTIR of JB_S01_B_F_1.

The raw spectra, expressed in percent transmittance ( %T), were acquired for each sample over the spectral range of 4000–370 cm⁻¹, covering the mid-infrared (MIR) region. Although the quality of the acquired spectra varied, the majority exhibited medium to strong signal spectra. The experimental spectrum for the JB_S01_B_F_1 sample, along with its corresponding spectral library match, is presented in Fig. 1. Further spectra are provided in the accompanying repository.

**Fig. 2:**
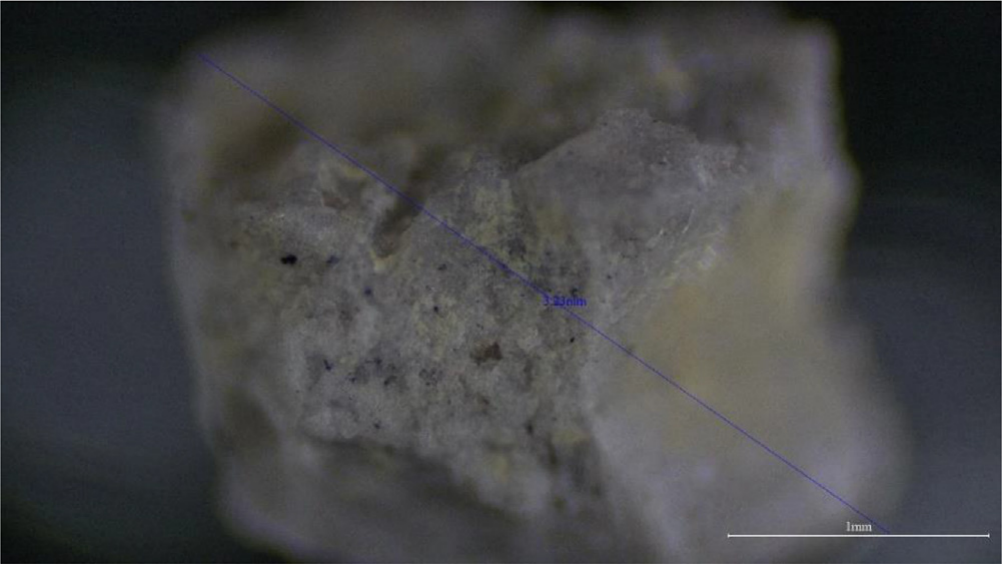
Microscopic image of JB_S01_B_F_1 collected from Juara Beach.

The current analysis revealed the presence of plastic particles including both microplastics (<5 mm) and macroplastics (>5 mm). Each size category was represented by a variety of colors and shapes (fragments and film), as shown by the microscopic images. The distribution and abundance of colors varying between locations. Particles from the Tulai site appeared to be the most numerous, with 22 particles recorded across the sampling sites. Meanwhile, Juara and Salang sites were moderately represented. In the present study, microscopic images of the observed microplastics were taken as shown in Fig. 2. The images of the particles are stored in data repository.

**Fig. 3:**
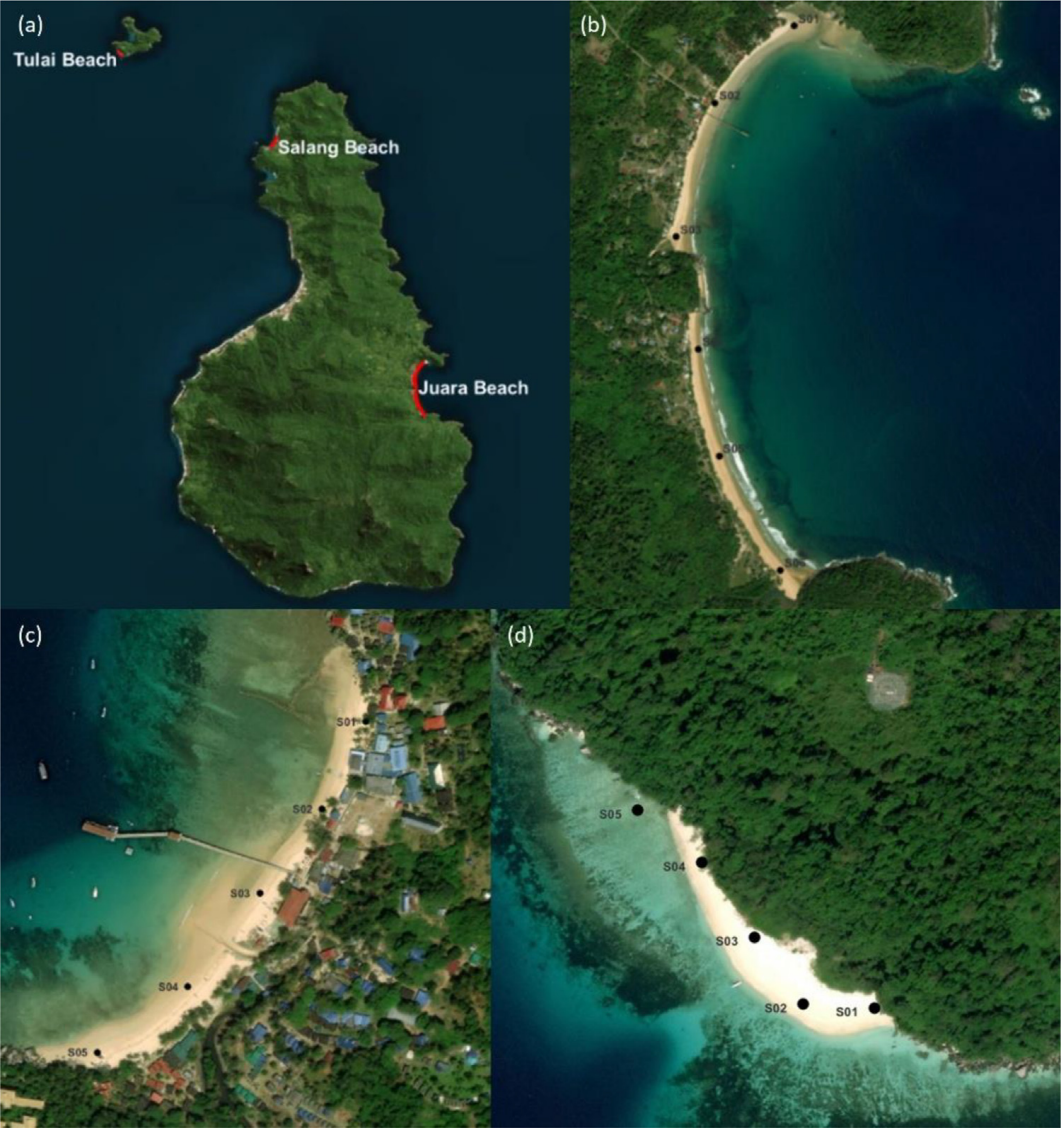
(a) Map of Tioman Island, Malaysia; (b) Juara Beach; (c) Salang Beach; (d) Map of Tulai Beach.

The maps provided illustrate the sampling locations for this study. Fig. 3(a) shows the overall map of Tioman Island, highlighting the general locations of the studied beaches. Fig. 3(b) presents a detailed view of Juara Beach, comprising six sampling sites (S01 to S06). Fig. 3(c) focuses on Salang Beach, where five sites were selected. Lastly, Fig. 3(d) shows Tulai Beach, with five sampling sites visited as well.

## 4 Experimental Design, Materials and Methods

### 4.1 Sampling

Microparticles were extracted from beach sediment samples through a sequential process involving sieving, density separation, and spectroscopic identification to ensure effective recovery and characterization [35,36]. Sediment samples were first extracted and sieved using cylindrical stainless-steel mesh sieves of 5.60 mm, 710 µm, and 212 µm to separate size fractions and isolate particles below 5 mm. The sieved fractions were subjected to density separation using clean seawater. This approach allowed for the systematic separation of particles into distinct size fractions, enhancing reproducibility across different sampling sites. Recovered particles were filtered and initially screened based on particle type, color, and size, followed by confirmation using FTIR spectroscopy to determine polymer composition.

**Fig. 4:**
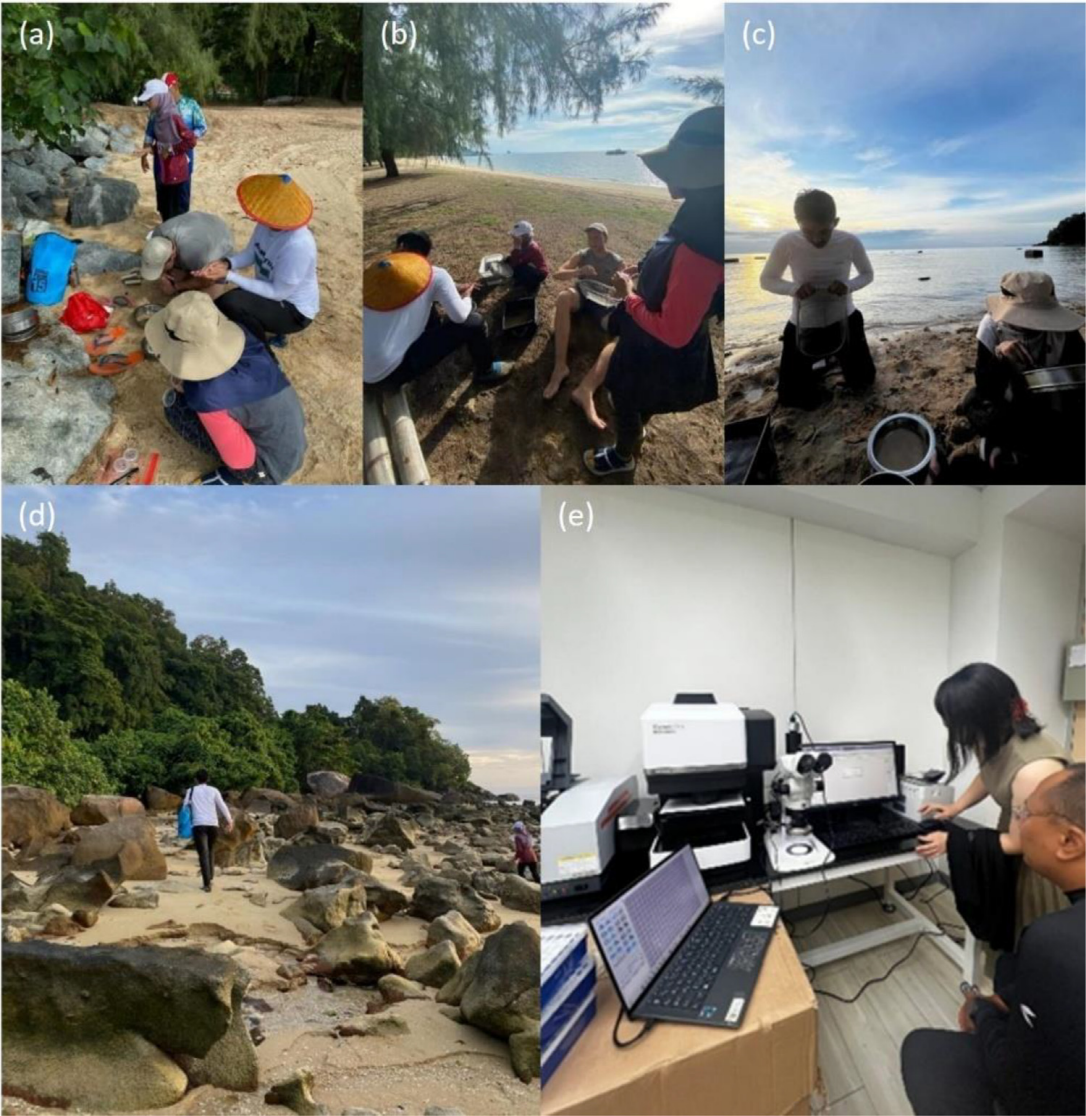
Sampling and data collection process. The authors are shown (a) collecting surface beach sediment samples, (b) sorting particles, and (c) performing multiple sieving steps on sandy beaches. (d) Landscape view of the beaches along the western edge of Tioman Island. (e) FTIR analysis of the collected particles.

These images highlight key moments from the sample collection process at three coastal sites: Juara, Tulai, and Salang. In Fig. 4(a), (b) and (c), the authors are collecting particles from sandy beaches using sieves. Fig. 4(d) shows some of the landscape of the beaches on the western edge of Tioman Island consists of natural rock beaches rather than typical sandy beaches. Fig. 4(e) shows the FTIR setup where authors analyzed the particles. This final phase was conducted with the strong support of Dr. Haruka Nakano from the Centre for Ocean Plastic Studies, Kyushu University.

### 4.2 Microscopic analysis

The samples collected from the sites were each placed in a unique code-labelled collection container of their own. They were then left to dry at room temperature in the laboratory before being transferred from the collection containers into labelled Eppendorf tubes. A transmitted light microscope (Olympus CX33), equipped with a camera and connected to a Dell monitor, was utilised to allow ease of viewing and detailed image capture of the samples. A ruler was used to calibrate the measurements using the 4 × objective lens. Each sample was taken out of the Eppendorf tube and placed onto a glass slide for observation. Microscopic images were captured after the measurement of the distance between the two furthest points of the sample according to the specific sample code and label. Each sample was transferred and analyzed one at a time to avoid confusion and misidentification.

To ensure reliability and reproducibility in microplastic microscopy analysis, all visual identification and particle counting were conducted by two analysts who underwent training sessions prior to the study. Although formal interlaboratory comparison (ILC) was not conducted during this phase, standard operating procedures (SOPs) laboratories were strictly followed throughout all measurements [37,38]. In addition, sample codes and microscopic images were recorded and archived to track analyst consistency.

### 4.3 FTIR analysis

FTIR spectral acquisition of the particles was performed using a Shimadzu IRAffinity-1S Fourier Transform Infrared (FTIR) spectrophotometer equipped with an attenuated total reflectance (ATR) accessory. Spectra were recorded in the mid-infrared region from 4000 to 370 cm⁻¹ with a spectral resolution of 4 cm⁻¹. Each spectrum was collected by averaging 32 scans. The instrument operated in %transmittance mode, with Happ-Genzel apodization and a mirror speed of 2.8. The internal beam and standard detector configuration were used, and no atmospheric correction or zero-filling was applied. Prior to each measurement, the ATR Dia/ZnSe was cleaned to prevent cross-contamination.

## Limitations

Despite rigorous protocols in sampling and data analysis, several limitations and potential contamination sources may have influenced the microplastic data collected from this particular field trip. Contamination could have arisen from airborne fibers or ambient plastic particles during field sampling especially in the absence of blanks. While 35 particles were analyzed, the dataset is biased toward particles >1 mm due to sieve cut-offs and color identification relied on visual assessment without validation with computer vision and artificial intelligence. Moreover, surface-only sampling may have excluded deeper particles, underscoring the need for integrated temporal and vertical profiling in future sampling.

## Ethics Statement

This research adheres to all applicable ethical standards and in compliance with institutional and regulatory ethical principles. No human and animal subjects were involved. All images used were self-acquired and not sourced from social media or other external platforms. All data were obtained in compliance with ethical guidelines.

## CRediT author statement

**Mohd Rashidi Abdull Manap**: Conceptualization, Funding, Methodology, Writing – original draft, Data curation, Supervision. **Muhammad Ibadurrahman bin Imizan**: Investigation, Visualization, Methodology, Writing – original draft. **Jannik Werner Dams**: Methodology, Visualization, Writing – original draft. **Norizah Abdul Rahman**: Funding, Writing – review & editing, Resources, Investigation. **Fathin Ayunni Zaiffira Waini**: Writing – original draft, Writing – review & editing. **Nur Hayatna Mukhni**: Writing – original draft, Writing – review & editing, Visualisation. **Wan Lutfi Wan Johari**: Writing – original draft, Writing – review & editing.

## Data Availability

Mendeley DataSupporting Dataset of FTIR, Microscopic Images, Sample Descriptions, and Study Map for Microplastic Analysis from Juara, Salang, and Tulai, Tioman Island, Malaysia (August 2024) (Original data).
